# The Effect of Online Parent Training With and Without Coaching Support on the Well‐Being of Families of Children With Neurodisabilities: A Randomized Controlled Trial

**DOI:** 10.1002/aur.70334

**Published:** 2026-07-30

**Authors:** Jeffrey McCrossin, Ali Filali‐Mouhim, Michael Ungar, Karen McEwan, Kathleen O'Grady, Donna Thomson, Parent Advisory Group, Patricia Lingley‐Pottie, Patrick McGrath, Lucyna M. Lach

**Affiliations:** ^1^ École de psychoéducation Université de Montréal Montréal Canada; ^2^ Centre intégré Universitaire de santé et de Services Sociaux du Centre‐Sud‐de‐L'île‐de‐Montréal Montréal Canada; ^3^ Resilience Research Centre Dalhousie University Halifax Canada; ^4^ IWK Health Centre Halifax Canada; ^5^ Simone de Beauvoir Institute Concordia University Montréal Canada; ^6^ Parent Advisory Group Canada; ^7^ CanChild McMaster University Hamilton Canada; ^8^ Strongest Families Institute Halifax Canada; ^9^ School of Social Work, McGill University Montréal Canada

**Keywords:** autism spectrum disorder, coaching support, family resilience, family well‐being, neurodisabilities, online intervention, parent training, randomized controlled trial

## Abstract

Families of children with neurodisabilities often face significant child behavioral challenges that can affect overall family functioning and well‐being. This randomized controlled trial (RCT) used family resilience theory as the investigative framework to examine the impact of an online parenting program designed to address child behavioral challenges. Parents of children with a range of neurodisabilities were randomized into three groups: a self‐directed online parenting program, the same program with coaching support, and a control group. Family well‐being was measured using the SCORE‐15 at baseline, 5, and 10 months. The primary analysis included a per‐protocol sample comprising self‐directed participants who completed ≥ 8 of 12 modules, coached participants who completed ≥ 8 modules and ≥ 8 coaching sessions, and all control‐group participants. Linear mixed models (LMMs) were used to analyze the repeated measures data, followed by standard linear regression models to assess the average intervention effect at each time point. Clinically significant changes were calculated via the reliable change index (RCI). Of the 454 families randomized, 422 participants had at least one available SCORE‐15 assessment (self‐directed: *n* = 140; coached: *n* = 139; control: *n* = 143). Descriptively, mean SCORE‐15 scores remained relatively stable in both intervention groups but increased over time in the control group (lower scores indicate better family functioning). Broader results from the full‐sample analysis are reported separately (publication forthcoming). The present analyses focus on the 248 participants who met the per‐protocol adherence criteria (self‐directed: *n* = 36; coached: *n* = 60; control: *n* = 152). Families that reported higher baseline family well‐being were associated with greater adherence to the intervention. Participants in both intervention groups demonstrated significant improvements in family well‐being relative to the control group. The coached version of the intervention demonstrated a more robust and persistent impact relative to the self‐directed version, particularly with respect to communication‐related subscales. Across both versions of the intervention 10%–28% of participants experienced reliable clinical change. The findings from our analysis suggest that both versions of the parenting program led to notable enhancements in aspects of family well‐being, with the coached group exhibiting particularly remarkable improvements. Our study highlights that parenting interventions can contribute to the well‐being of the entire family system, extending beyond individual child and parent outcomes typically addressed by these programs.

## Summary

This study looked at how effective online parenting programs are for families of children with neurodisabilities like autism and cerebral palsy. Some parents had extra help through coaching, while others did not. These programs taught parents how to handle challenging child behaviors and improve family life. The study found that while both versions of the program had some positive impact, families who received extra coaching seemed to benefit more, especially in how they communicate and solve problems together. Our results suggest that with the right support, families can get better at dealing with the challenges that come with parenting children with these conditions. Our hope is that these findings will help create better programs for families who need extra support.

## 1 Introduction

Families of children with neurodisabilities, such as autism spectrum disorder (ASD), fetal alcohol spectrum disorder (FASD), cerebral palsy, and other brain‐based developmental disabilities (Morris et al. 2013), often experience comorbid behavioral challenges including aggression, self‐injurious behavior, anxiety, hyperactivity, and difficulty with emotional regulation. Some estimates of the prevalence of this comorbidity range from 30% to 64% depending on the disability (Dekker et al. 2002; Emerson 2003; Lach et al. 2009; Micai et al. 2023; Petrenko 2013; Petrou et al. 2018). Parents and other caregivers (hereafter referred to as “parents” to simplify the text and reflect our focus in this study on enhancing parenting skills) often seek support for managing these behavioral challenges due to their impact on the child and the whole family (McCrossin and Lach 2023a). Existing interventions are often costly and difficult to access. Furthermore, there is limited research on their impact on the family unit (McCrossin and Lach 2023b; Weeland et al. 2021). The present study examines family well‐being as an outcome in the context of a large, randomized controlled trial (RCT) of an online parenting program tailored to families of children living with neurodisabilities in Canada.

Parenting programs have become a prevalent strategy for addressing behavioral challenges of children who have neurodisabilities (Bearss et al. 2015; Grenier‐Martin et al. 2022; McCrossin et al. 2023; Postorino et al. 2017; Tarver et al. 2019). These programs are not only significant for their public health impact due to their potential for scalability (Sanders et al. 2021) but also for the foundational role they play in recognizing and supporting the child's right to a promising start in life. Enhancing parents' skills through these programs can solidify the groundwork for optimal child development and overall family well‐being (Weeland et al. 2021).

Taking a parent‐focused approach, rather than one that requires direct intervention with the child by a therapist, acknowledges the systemic effects of behavioral challenges within the family unit and affirms the integral role of parents in managing the family and child's everyday life (Factor et al. 2019; Karst and Van Hecke 2012; Sikora et al. 2013). Parenting programs, regardless of the child population being supported, typically aim to improve behavior management skills, communication techniques, and environmental adaptations that promote positive parent–child interactions (Kaminski et al. 2008; Petrenko 2013). However, the range of programs is highly diverse, differing in their theoretical underpinnings, targeted disabilities, methods of delivery, program structure, and educational content (Dababnah and Parish 2016; McCrossin et al. 2023; Postorino et al. 2017).

Since parenting programs are typically offered in response to parental concerns over child behaviors, clinical trials of these programs have traditionally focused on improving child behavior and neglected to account for family‐level outcomes (Dykens 2015; McCrossin et al. 2023; McCrossin and Lach 2023a, 2023b). However, there are a host of reasons why outcomes at the level of the family unit should be included in this field of research. For example, in addition to child emotional health, family well‐being remains a primary health concern for parents in this population (Allard et al. 2014). Furthermore, since most parenting programs involve little‐to‐no direct contact between the program facilitator and the child, families are highly involved in the application of the interventions learned over the course of the program. The impact of the program is therefore likely to extend beyond child behavior to affect the entire family context. In addition to the transfer of knowledge and skills from parents to other caregivers in the family, interventions are often applicable to other children in the home, including those who are neurotypical. Since interventions are intended to improve child behavior and parental responses, one could reasonably expect that an outcome from these programs would be some improvement for the family unit as well. The issue of including family‐level outcomes has been raised by many in the literature (Dykens 2015; Factor et al. 2019; Kuhaneck et al. 2015; Sikora et al. 2013). However, identifying what aspects of the family to measure and how to measure them is complex (Bailey et al. 2012; Lach 2013; McCrossin and Lach 2023b).

A range of self‐report family‐level outcome measures exist both for the general population (Alderfer et al. 2008) and specifically for families of children with neurodisabilities (Ketelaar et al. 2017), requiring researchers to make a choice regarding how to measure change in the family in the context of parenting programs. Among the most commonly used is the Family Assessment Device (Epstein et al. 1983). There are various editions of this measure, but all are intended to give a general snapshot of family functioning at a given point in time in relation to key family processes such as communication, behavior control, and role or task assignment. Others, such as the Beach Centre Family Quality of Life Scale (Hoffman et al. 2006), were developed specifically with families of persons with disabilities in mind. These measures usually include specific items that commonly resonate with this specific population; however, they often center on the person with the disability, the impact the disability has on the family, or how well the family rallies to support the person with the disability, rather than more general family processes that place the family unit at the center of focus. Given the range of reasonable options for family‐level outcome measures, researchers must first identify a construct to measure, such as general family functioning or the impact of the disability on the family, and provide a rationale for the fit of the measure with respect to an underlying theoretical model that guides their decision.

In the present study, we use family resilience theory (Maurović et al. 2020; McCubbin and Patterson 1983; Ungar 2019; Walsh 2021) to guide our investigation. Family resilience refers to the process by which families do well in the face of adversity (Ungar 2010). Considering this construct as a process, rather than a state or trait, we can identify risk and protective factors that contribute to family well‐being. Family resilience theory has been increasingly invoked to describe the experience of families of children with neurodisabilities (for a review see Gunty 2020; Leone et al. 2016). Many of these recent research applications of family resilience theory use McCubbin and Patterson's (1983) Double ABCX model to create statistical models identifying contributions of family stressors, belief systems or perceptions, and internal and external family resources on family well‐being. Through this family resilience theoretical lens, parenting programs can be viewed as fostering family resilience which facilitates positive effects on family well‐being. These programs impact parenting processes such as parental self‐efficacy, identification of and access to internal resources, and provide knowledge, skills training, and connections to multiple forms of social and instrumental support that are not limited to aspects related to childhood neurodisabilities (Masten and Palmer 2019). Therefore, for this study we hypothesized that these intervention‐related changes in parenting processes would have a positive impact on the general functioning of the family.

The present article presents an exploratory analysis of family outcome variables drawn from an extensive dataset from a broader study (publication forthcoming; see protocol registration at clinicaltrials.gov, identifier: NCT03835689) examining child and parent outcomes of an online parenting program. The outcome variables used in the present study were not identified as primary or secondary outcomes in the original study. Through a per protocol approach, our investigation aims to assess the impact of the parenting program on family well‐being among families with children who have neurodisabilities and exhibit challenging behaviors. Specifically, we investigate how parents report change in family well‐being over the course of the coached and uncoached versions of the parenting program when compared to a control group.

## 2 Methods

### 2.1 Protocol Registration and Ethics

This trial was registered with ClinicalTrials.gov (Identifier: NCT03835689). The research project was approved by the Review Ethics Boards of the IWK Health Centre (#1023970) and McGill University (#107–0719).

### 2.2 Participants

#### 2.2.1 Recruitment

Participants were recruited from April 2019 until October 2021 through an existing Canada‐wide database of parents who participated in a previous study and agreed to be contacted again in the future, neurodevelopmental clinics and service providers, disability‐specific research registries, other venues where parents of children with neurodisabilities have connections and through networks of the parent advisory group (PAG) involved with the study (e.g., Facebook groups and other forms of social media). The maximum required sample size for the broader study was set for 450 participants, determined based on a planned intent‐to‐treat analysis for the individual child and caregiver outcomes. However, for the present independent exploratory analysis of the family outcome, a per‐protocol analysis limited the sample size to 248 participants.

#### 2.2.2 Inclusion Criteria

To be eligible for the study, participants had to be a parent of a child with a neurodisability (e.g., ASD, cerebral palsy, epilepsy, global developmental delay, down syndrome, FASD, severe learning disability, or any diagnosis that influences how the child gets around, communicates, or uses their memory), as reported by the parent. Children were required to meet a minimum score of 16 on the Total Difficulties Score of the Strengths and Difficulties Questionnaire (Goodman 2001) for those aged 3–4 years, and a minimum score of 17 for those aged 5–17 years. Participants had to be the primary caregiver for at least 6 months prior to the study and intend to continue to be the primary caregiver for at least the duration of the study. Participants also had to be able to read, write, and understand English; have access to a telephone and an internet‐connected device; live in Canada; and commit to engagement in the program (e.g., attending weekly coaching calls for the coached condition and having a Facebook account to interact with the parent‐to‐parent support component).

#### 2.2.3 Exclusion Criteria

Potential participants were excluded if parents reported that (a) their child was unable to understand everyday language and simple, one‐step instructions from parents, or to communicate their basic needs for food or comfort; (b) the parent or child had unmanaged symptoms of psychosis, schizophrenia, bipolar disorder, or major depression; (c) the child was at imminent risk of serious harm to self or others; (d) the child regularly engaged in moderate‐to‐severe self‐injurious behavior; (e) the child had attempted suicide in the previous 6 months; (f) or the parent had participated in another parenting program within the past 6 months.

### 2.3 Intervention

The present study involves the Strongest Families Parents Empowering Neurodiverse Kids (SFND) positive parenting program which is an adaptation of Strongest Families Institute's (SFI) Parents Empowering Kids program that has been shown effective in clinical trials (McGrath et al. 2011; Olthuis et al. 2018; Sourander et al. 2016, 2018), based on a cognitive–behavioral approach for families of children with a range of challenges including those related to conduct, attention‐deficit/hyperactivity disorder (ADHD), and oppositional defiance. The programs are delivered online and are self‐directed, with or without support from a paraprofessional trained in SFI's telephone coaching protocols on parent training. As such, the program offers a low‐intensity intervention suitable for delivery on a large scale due to its low cost and accessibility relative to in‐person therapy, and flexibility due to the online, self‐paced format.

SFI coaches undergo intensive training during their first 6 months, with a focus on background information related to the specific conditions and comorbidities; SFI skill‐based program materials (e.g., program content, skill‐demonstration media, prescribed telephone call protocols); SFI's IRIS software platform navigation and documentation protocols; and telephone call training with a supervisor (Lingely‐Pottie and McGrath 2023; Lingley‐Pottie and McGrath 2016). Coach calls are recorded and graded, regardless of coach tenure. Ongoing telephone call evaluation monitors coach competency in key performance areas, with additional training provided if thresholds are not consistently achieved, including adherence to risk management protocols.

The program includes 11 modules plus a booster session focused on principles of positive parenting, covering content such as noticing and giving attention to desirable behavior, planned ignoring of undesirable behavior (e.g., whining or complaining), problem solving, reward systems, emotion regulation, and generalizing behavior management to different contexts, such as school or daycare. The program includes content specific to the population of interest, including an introductory module and examples and graphics depicting families of children with neurodisabilities. Furthermore, the program content is tailored to each family and allows for parents to identify and apply goals to their family circumstances, with regular prompts providing opportunities to review and elaborate on these goals from module to module.

For this study, two variations of the SFND program were evaluated. The SFND‐self group engaged with a self‐directed version of the parenting program modules, while the SFND‐c group received the same self‐directed materials, supplemented by weekly 45‐min group coaching sessions conducted via phone by trained paraprofessionals. The purpose of the coaching sessions was to equip parents with positive parenting skills, support parents to stay on track with intervention goals, and successful implementation of the skills within their activities of daily living. Parents in both intervention conditions had access to a closed Facebook group with other parents in the study. Posts in the social media component of the study were consistent with the content of the intervention; the group was monitored and facilitated by two parents (one for each intervention group) who were paid a stipend for their engagement. The facilitator monitored the group to set the tone for discussion, acknowledge questions and comments, and posted content related to the program modules. Parents had access to the group until 6 months after the last control group participants were provided access.

### 2.4 Design and Procedure

This was a three‐arm, longitudinal RCT. After meeting eligibility criteria, participants were randomly assigned to a self‐directed module‐based parent‐training program (SFND‐self), a coached version of the same program (SFND‐c), or a control group receiving access to a static resource website containing information and resources for parents of children with neurodisabilities (e.g., book titles, other websites, and links to relevant organizations; https://www.childhooddisability.ca/). Parents assigned to the control group were given access to the self‐directed intervention following completion of their participation in the study. Measurements were collected at three time points: baseline (T1), as well as 5 months (T2), and 10 months (T3) post randomization. Study activities were completed between April 2019 and September 2022. Participants received a $50 gift card or cheque after completing measurements at baseline and after each follow‐up assessment. A parent advisory group (PAG) was involved in every stage of the study including adapting the original intervention, reviewing the modules, recruitment, research design, measurement selection and manuscript publication. The present article reports findings from a separate analysis of family well‐being, which complements the primary and secondary analyses of child and parent outcomes conducted by the principal investigators (LL and PM) of the broader study.

Demographic questionnaires and assessments were completed online using REDCap data collection and management software hosted at the Women and Children's Health Research Institute at the University of Alberta (Harris et al. 2009; Women and Children's Health Research Institute 2022). Parents reported on their child's functional impairment across several domains, including vision, hearing, speech, locomotion, and learning. These responses were computed to generate an overall child function score, representing the cumulative number of functional limitations across all areas. Additionally, parents noted any developmental diagnoses their children had received. For analysis, a hierarchical model classified these diagnoses, giving precedence to certain conditions. When reported, ASD would override other conditions in classification. If ASD was absent but cerebral palsy was reported, it would determine the group. This method also prioritized FASD and intellectual disability. Diagnoses not fitting these categories were labeled “other disability,” capturing the diverse range of less common conditions in our sample, including Down syndrome, epilepsy, global developmental delay, spina bifida, and attention‐deficit/hyperactivity disorder if not paired with a learning disability. The final conditions included in the disability groups are consistent with the established literature (Miller, Gardiner, and Rosenbaum 2023).

### 2.5 Randomization Methods

Participants were randomly assigned to one of three study groups using REDCap's randomization feature with concealed block sizes to ensure research staff blindness. To ensure an even distribution of children with differing care needs, randomization was stratified by parent‐reported cognitive functioning (“no or some difficulty” or “a lot of difficulty”) in REDCap. This stratification ensured comparable baseline cognitive challenge across arms, reducing the risk that any group would be over‐ or under‐represented by children with higher support needs.

### 2.6 Measure of Family Well‐Being

Following consultation with the PAG, the family‐level outcome measure chosen was the Systemic Clinical Outcome and Routine Evaluation (SCORE‐15) (Carr and Stratton 2017; Stratton et al. 2014). This measure assesses parents' perceptions of their family's overall functioning. Our focus was to determine how the parenting program influenced general family functioning, rather than functioning specifically related to their child's disability. This self‐report measure consists of 15 positively and negatively worded Likert scale items designed to capture family functioning and therapeutic change in families (see https://www.aft.org.uk/page/score). The SCORE‐15 produces a total score and three sub‐scales inclusive of *Family Strengths and Adaptability* (e.g., “Each of us gets listened to in our family”), *Overwhelmed by Difficulties* (e.g., “We find it hard to deal with everyday problems”), and *Disrupted Communication* (e.g., “People often don't tell each other the truth in my family”). The potential total score for this assessment ranges from 15 to 75, with a higher total score indicating a more negative evaluation of the respondent's family. The SCORE‐15 has been shown to have good internal consistency, test–retest reliability, and responsiveness to change over three to 5 months in the context of therapeutic interventions (Hamilton et al. 2015).

### 2.7 Statistical Analysis

The first two authors conducted the data analysis for the present study independently of the analysis of the main study. First, the dataset was cleaned to exclude records that had not completed randomization. Participants were then classified into one of three groups based on the intervention they received: SFND‐c, SFND‐self, and the control group. Descriptive statistics were computed for all demographic characteristics and covariates (Tables 1, 2, 3). For continuous variables, intergroup differences were assessed using the Kruskal–Wallis rank test, whereas categorical variables were analyzed with Pearson's Chi‐squared test. When expected frequencies were less than five for categorical variables, Fisher's Exact test was employed as an alternative.

**TABLE 1:** Summary of demographic statistics for child‐centered variables.

Variables	Categories	SFND‐c (*n* = 60)	SFND‐self (*n* = 36)	Control (*n* = 152)	Total (%)	*p*
Child gender	Female	12 (20.0%)	10 (27.8%)	36 (23.7%)	58 (23.4%)	0.68^a^
	Male	48 (80.0%)	26 (72.2%)	116 (76.3%)	190 (76.6%)	
Disability group	ADHD and learning disability	3 (5.0%)	2 (5.6%)	9 (5.9%)	14 (5.6%)	0.95^b^
	Autism spectrum disorder	38 (63.3%)	25 (69.4%)	98 (64.5%)	161 (64.9%)	
	Cerebral palsy	2 (3.3%)	1 (2.8%)	5 (3.3%)	8 (3.2%)	
	Fetal alcohol spectrum disorder	2 (3.3%)	2 (5.6%)	11 (7.2%)	15 (6.0%)	
	Intellectual disability	6 (10.0%)	4 (11.1%)	11 (7.2%)	21 (8.5%)	
	Other disability	9 (15.0%)	2 (5.6%)	18 (11.8%)	29 (11.7%)	
Type of behavior	Externalizing	14 (23.3%)	9 (25.0%)	35 (23.0%)	58 (23.4%)	0.09^b^
	Internalizing	13 (21.7%)	8 (22.2%)	26 (17.1%)	47 (19.0%)	
	Internalizing and externalizing	29 (48.3%)	19 (52.8%)	91 (59.9%)	139 (56.0%)	
	None of the above	4 (6.7%)	0 (0.0%)	0 (0.0%)	4 (1.6%)	
Child age	Minimum	3.00	4.00	3.00		0.41^c^
	Maximum	14.00	14.00	14.00		
	Mean	7.75	7.67	8.24		
	SD	3.52	2.79	3.17		
Child function	Minimum	0.00	0.00	0.00		0.6^c^
	Maximum	4.00	3.00	4.00		
	Mean	1.78	1.64	1.85		
	SD	1.17	1.07	1.03		

*Note:* See methods section for details on development of disability group and child function calculations.

Abbreviations: SFND‐c = strongest families neurodevelopmental—coached intervention; SFND‐self = strongest families neurodevelopmental—self‐directed intervention.

^a^ Pearson's chi‐square test.

^b^ Fisher's exact test.

^c^ Kruskal–Wallis test.

**TABLE 2:** Summary of demographic statistics for parent‐centered variables.

Variables	Categories	SFND‐c (*n* = 60)	SFND‐self (*n* = 36)	Control (*n* = 152)	Total (%)	*p*
Parent ethnicity	African origins	1 (1.8%)	0 (0.0%)	1 (0.7%)	2 (0.9%)	0.59^b^
	Caribbean/Latin/Central/South American Origins	3 (5.4%)	0 (0.0%)	3 (2.1%)	6 (2.6%)	
	Central Asian and Middle Eastern Origins	0 (0.0%)	0 (0.0%)	2 (1.4%)	2 (0.9%)	
	East and South Asian Origins	4 (7.1%)	2 (6.1%)	10 (6.9%)	16 (6.8%)	
	European Ancestry	40 (71.4%)	26 (78.8%)	105 (72.4%)	171 (73.1%)	
	North American Indigenous Origins	0 (0.0%)	0 (0.0%)	6 (4.1%)	6 (2.6%)	
	Other	4 (7.1%)	2 (6.1%)	14 (9.7%)	20 (8.5%)	
	Prefer not to say	4 (7.1%)	3 (9.1%)	4 (2.8%)	11 (4.7%)	
Parent gender	Female	59 (98.3%)	35 (97.2%)	143 (94.1%)	237 (95.6%)	0.54^b^
	Male	1 (1.7%)	1 (2.8%)	9 (5.9%)	11 (4.4%)	
	Not specified	0 (0.0%)	0 (0.0%)	0 (0.0%)	0 (0.0%)	
Parent education	Associate/doctorate/professional degree	7 (11.7%)	0 (0.0%)	11 (7.3%)	18 (7.3%)	0.02^b^
	Master's degree	15 (25.0%)	10 (27.8%)	20 (13.3%)	45 (18.3%)	
	Bachelor's degree	18 (30.0%)	6 (16.7%)	64 (42.7%)	88 (35.8%)	
	Some college (no degree)	8 (13.3%)	10 (27.8%)	18 (12.0%)	36 (14.6%)	
	Occupational/technical/vocational program	9 (15.0%)	5 (13.9%)	24 (16.0%)	38 (15.4%)	
	High school graduate or GED	1 (1.7%)	3 (8.3%)	7 (4.7%)	11 (4.5%)	
	Unknown/not specified	2 (3.3%)	2 (5.6%)	6 (4.0%)	10 (4.1%)	
Parent age	Minimum	27.00	28.00	27.00		0.44^c^
	Maximum	59.00	55.00	66.00		
	Mean	42.65	40.89	42.82		

Abbreviations: SFND‐c = Strongest Families Neurodevelopmental – coached intervention; SFND‐self = Strongest Families Neurodevelopmental—self‐directed intervention.

^a^ Pearson's chi‐square test.

^b^ Fisher's exact test.

^c^ Kruskal–Wallis test.

**TABLE 3:** Summary of demographic statistics for family‐centered variables.

Variables	Categories	SFND‐c (*n* = 60)	SFND‐self (*n* = 36)	Control (*n* = 152)	Total (%)	*p*
Household income	Over $200,000	4 (6.7%)	2 (5.6%)	5 (3.3%)	11 (4.5%)	0.56^b^
	$100,000–$199,999	19 (31.7%)	13 (36.1%)	43 (28.5%)	75 (30.4%)	
	$75,000–$99,999	14 (23.3%)	5 (13.9%)	30 (19.9%)	49 (19.8%)	
	$50,000–$74,999	9 (15.0%)	6 (16.7%)	29 (19.2%)	44 (17.8%)	
	$35,000–$49,999	3 (5.0%)	3 (8.3%)	12 (7.9%)	18 (7.3%)	
	$25,000–$34,999	2 (3.3%)	1 (2.8%)	12 (7.9%)	15 (6.1%)	
	$15,000–$24,999	2 (3.3%)	1 (2.8%)	3 (2.0%)	6 (2.4%)	
	Under $15,000	0 (0.0%)	4 (11.1%)	5 (3.3%)	9 (3.6%)	
	Not specified/unknown	7 (11.7%)	1 (2.8%)	12 (7.9%)	20 (8.1%)	
Family circumstances	Additional family challenges	26 (43.3%)	17 (47.2%)	75 (49.3%)	118 (47.6%)	0.73^a^
	Not applicable/not specified	34 (56.7%)	19 (52.8%)	77 (50.7%)	130 (52.4%)	
Household composition	Birth parents	48 (80.0%)	22 (61.1%)	104 (68.4%)	174 (70.2%)	0.15^b^
	Parent and step‐parent	4 (6.7%)	2 (5.6%)	6 (3.9%)	12 (4.8%)	
	Single parent	3 (5.0%)	6 (16.7%)	23 (15.1%)	32 (12.9%)	
	Foster/adoptive/legal guardian	2 (3.3%)	4 (11.1%)	13 (8.6%)	19 (7.7%)	
	Not specified/other	3 (5.0%)	2 (5.6%)	6 (3.9%)	11 (4.4%)	
Province	BC	15 (25.0%)	5 (13.9%)	31 (20.4%)	51 (20.6%)	0.01^b^
	NL	1 (1.7%)	5 (13.9%)	7 (4.6%)	13 (5.2%)	
	NS	1 (1.7%)	2 (5.6%)	12 (7.9%)	15 (6.0%)	
	NT	0 (0.0%)	3 (8.3%)	1 (0.7%)	4 (1.6%)	
	ON	39 (65.0%)	18 (50.0%)	81 (53.3%)	138 (55.6%)	
	PE	0 (0.0%)	2 (5.6%)	1 (0.7%)	3 (1.2%)	
	QC	2 (3.3%)	1 (2.8%)	15 (9.9%)	18 (7.3%)	
	SK	2 (3.3%)	0 (0.0%)	4 (2.6%)	6 (2.4%)	
Number of dependents	Minimum	1.00	1.00	1.00		0.15^c^
	Maximum	5.00	6.00	6.00		
	Mean	2.32	2.03	2.23		
	SD	0.85	1.06	1.03		

Abbreviations: SFND‐c = strongest families neurodevelopmental—coached intervention; SFND‐self = strongest families neurodevelopmental—self‐directed intervention.

^a^ Pearson's chi‐square test.

^b^ Fisher's exact test.

^c^ Kruskal–Wallis test.

The main analysis of the family well‐being measure involved a series of linear mixed models (LMMs) and standard linear regression models based on a per‐protocol analysis whereby data for participants were included if they completed at least eight out of 12 online modules for the SFND‐self condition or eight modules plus eight coaching sessions for the SFND‐c condition. We compared key sociodemographic characteristics and baseline family‐wellbeing scores between participants who did versus did not adhere to the protocol, using Kruskal–Wallis tests for continuous variables and Fisher's exact tests for categorical variables. All analyses for the study were performed using the statistical software R version 4.3.1 (R Core Team, 2021). The LMMs were implemented using the R package “lmerTest.”

#### 2.7.1 Linear Models

The use of linear models is appropriate for longitudinal RCTs with repeated measures completed by the same individuals, as they account for inherent clustering as well as missing data (Garson 2013). Unlike repeated measures analysis of variance (ANOVA) which requires complete datasets, mixed models handle missing data and dropouts well with maximum likelihood estimation, especially in the context of longitudinal designs, by retaining all the subjects who have data for at least one time point (Chakraborty and Gu 2009; Peters et al. 2012). An LMM was used to assess the time by intervention interaction, with a random intercept included to address the non‐independence of within‐individual observations.

To assess the impact of the intervention at each time point *without* accounting for the repeated measures (i.e., without random effects/intercepts), and to further investigate the confounding effects of other parameters, a series of standard nested linear models were used (see Appendix I for model equations). These models were organized into three sets: the Child Model (variables related to the child), the Child and Parent Model (variables related to both the child and parent), and the Full Family Model (variables related to the child, parent, and family). Each observation was treated as independent in these analyses. To address issues of multicollinearity several covariables were removed, including parent ethnicity from the Child and Parent Model and parental structure (i.e., biological, adoptive, step‐parent, etc.), household income, and number of dependents in the home from the Full Family Model.

We performed ANOVA to compare the fit of the models, specifically comparing each model against the baseline one. For each comparison, the *F*‐test was used to determine if there were statistically significant differences in the fit of the models.

#### 2.7.2 Reliable Change Scores

Lastly, we calculated reliable change index (RCI; Equation 1) (Blampied 2022; Jacobson and Truax 1991) scores to assess the significance of changes in scores over time for participants across different intervention groups at 5 and 10 months. Reliability coefficients, as reported by Hamilton et al. (2015), were applied to the *Total Score* and the *Family Strengths and Adaptability*, *Overwhelmed by Difficulties*, and *Disrupted Communication* subscales, with values of 0.91, 0.82, 0.91, and 0.85, respectively. The standard error of measurement (SEM) was calculated using the standard deviation of baseline scores and the aforementioned reliability coefficients. The SEM was then used to determine the RCI, indicating a clinically significant change if the index exceeded a threshold of 1.96 reflecting a 95% confidence interval.

(1) $$\mathrm{RCI}=\frac{\text{Scor}e_{\text{followup}}-\text{Scor}e_{\text{baseline}}}{SD_{\text{baseline}}\cdot \sqrt{2\cdot \left(1-\text{reliability}\right)}}$$

## 3 Results

**FIGURE 1:**
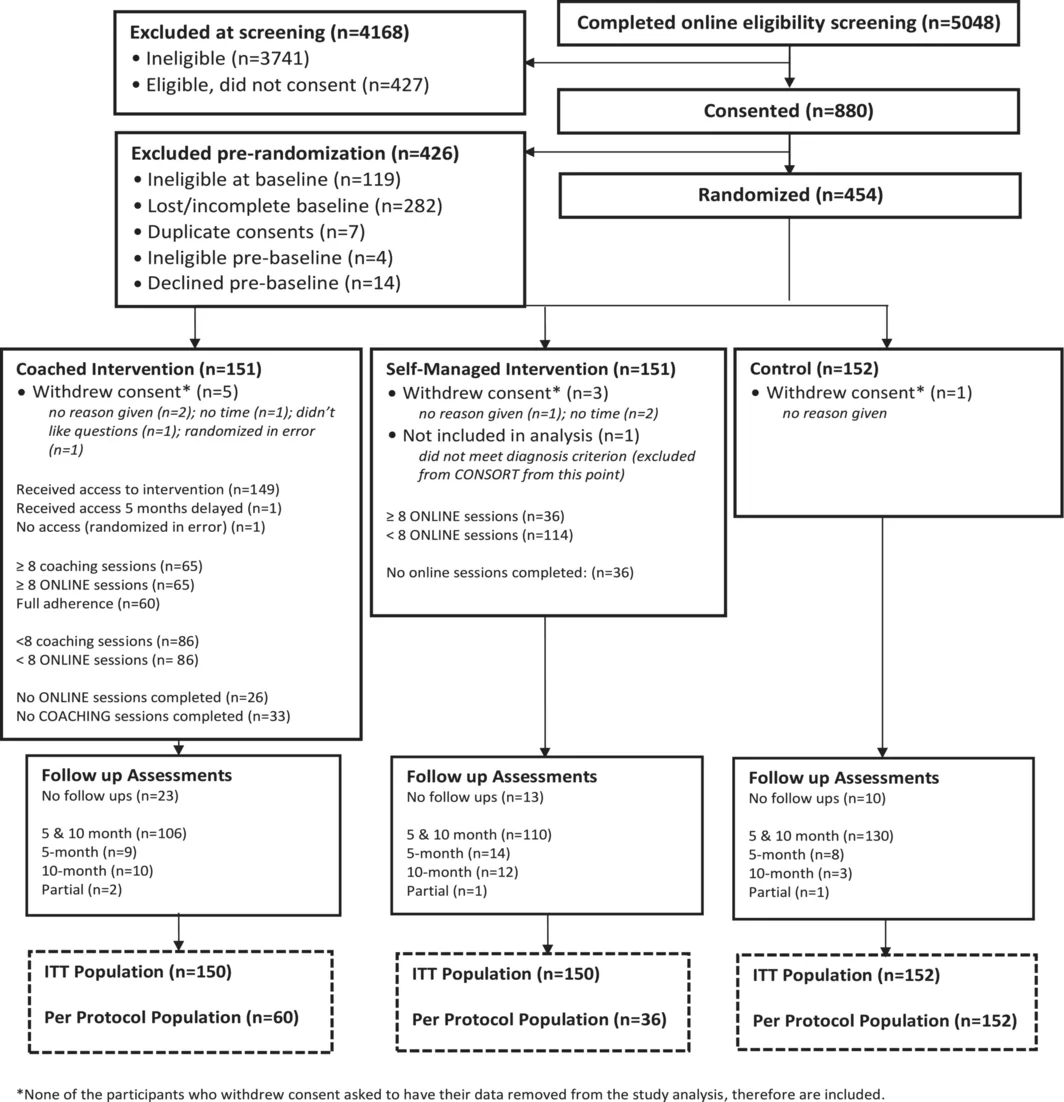
CONSORT flow diagram of participant progress through the phases of the randomized trial including screening, allocation, follow‐up, and analysis.

The CONSORT diagram (Figure 1) illustrates the progression of participants through the study. Of the 5477 individuals who completed the online screening, 880 provided informed consent. After baseline assessment and the exclusion of ineligible participants, 454 were randomized equally across the three study arms (SFND‐c, *n* = 151; SFND‐self, *n* = 151; control, *n* = 152). For the primary per‐protocol analysis, 248 families met the adherence criteria or were retained as controls (SFND‐c, *n* = 60; SFND‐self, *n* = 36; control, *n* = 152). Within the SFND‐c group, 60 participants adhered to the full protocol by completing at least 8 out of 12 of both the online and coaching components of the program, while 26 did not complete any online modules and 33 did not complete any coaching sessions. In the SFND‐self group, 36 participants adhered to the protocol by completing over 80% of the modules, while another 36 completed no sessions.

Before applying adherence criteria, 422 participants had at least one non‐missing SCORE‐15 total score (SFND‐c: *n* = 139; SFND‐self: *n* = 140; control: *n* = 143). Baseline SCORE‐15 total scores were available for 338 participants, 5‐month scores for 322 participants, and 10‐month scores for 351 participants. Descriptively, mean SCORE‐15 total scores were 30.27 at baseline, 29.48 at 5 months, and 30.48 at 10 months in the SFND‐c group; 30.85, 29.28, and 30.06 in the SFND‐self group; and 31.85, 32.19, and 32.52 in the control group, respectively, with lower scores indicating better family functioning. Given that the present manuscript focuses on exploratory family well‐being outcomes using a per‐protocol approach, the analyses reported below are based on the per‐protocol sample. Findings from the broader trial, including all available participants, are reported separately (publication forthcoming).

Demographic data are presented in Tables 1, 2, 3. Responding parents predominantly identified as female (95.6%), were of European ancestry (73%), and reported having some education beyond high school (86.7%). Child ages ranged from 3 to 14 years (*M* = 8.0, SD = 3.2) with 23.4% being identified by their parent as female. About 70% of the families involved in the study reported having a household composition that included the birth parents, with a range of one to six dependents (*M* = 2.2, SD = 1.0). Parent ages ranged from 27 to 66 (*M* = 42.5, SD = 6.8). Most families reported being residents of Ontario (55.7%) or British Columbia (20.6%) with the remaining participants living in one of six other provinces or territories. More than half of the respondents (54.4%) reported a household income of greater than $75,000. Nearly half of the families (47.6%) reported additional family challenges that they felt affected their ability to care for their child (e.g., employment loss, immigration, other family member with a medical condition). Child behavior concerns were reported as mostly a combination of both internalizing and externalizing problems (56.1%). Most parents reported that their child had a diagnosis of ASD (64.9%). The combined average number of child functional impairments reported across groups was 1.80 (SD = 1.07; range = 0–4).

In assessing the baseline characteristics of our sample across the intervention groups, we employed a series of statistical tests to analyze child, parent, and family‐level variables across intervention and control groups. For the continuous variables, Kruskal–Wallis tests indicated no significant differences in the distributions of these variables. For the categorical variables, no significant differences between groups were found using Pearson's chi‐squared and Fisher's exact tests except for the province of residence (*p* = 0.01) and parent education (*p* = 0.02), which we adjusted for in our models. Comparing families who did versus did not adhere to the intervention revealed no significant differences in age, income, household composition, child functioning, or other demographic factors except for parent ethnicity (*p* = 0.03). Specifically, 68.8% of adherent participants reported European ancestry versus 58.3% of non‐adherents. However, participants that adhered scored significantly better on the *Total Score* of the family well‐being measure at baseline with a mean of 29.0 (SD = 9.85) in participants that adhered versus 31.4 (SD = 9.43) in those that did not (*p* = 0.02).

**FIGURE 2:**
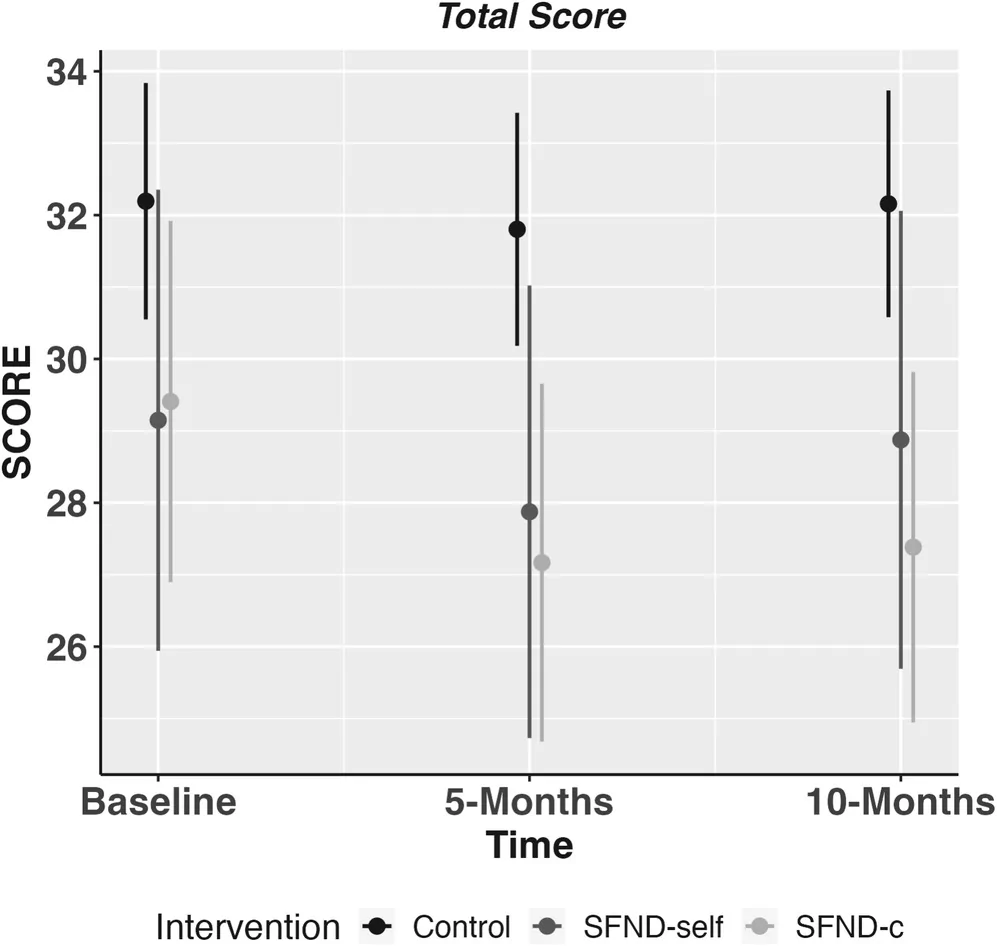
Plot of time*intervention interaction from interaction model for total score on family well‐being measure. Abbreviations: SFND‐c = strongest families neurodevelopmental—coached intervention; SFND‐self = strongest families neurodevelopmental—self‐directed intervention.

**FIGURE 3:**
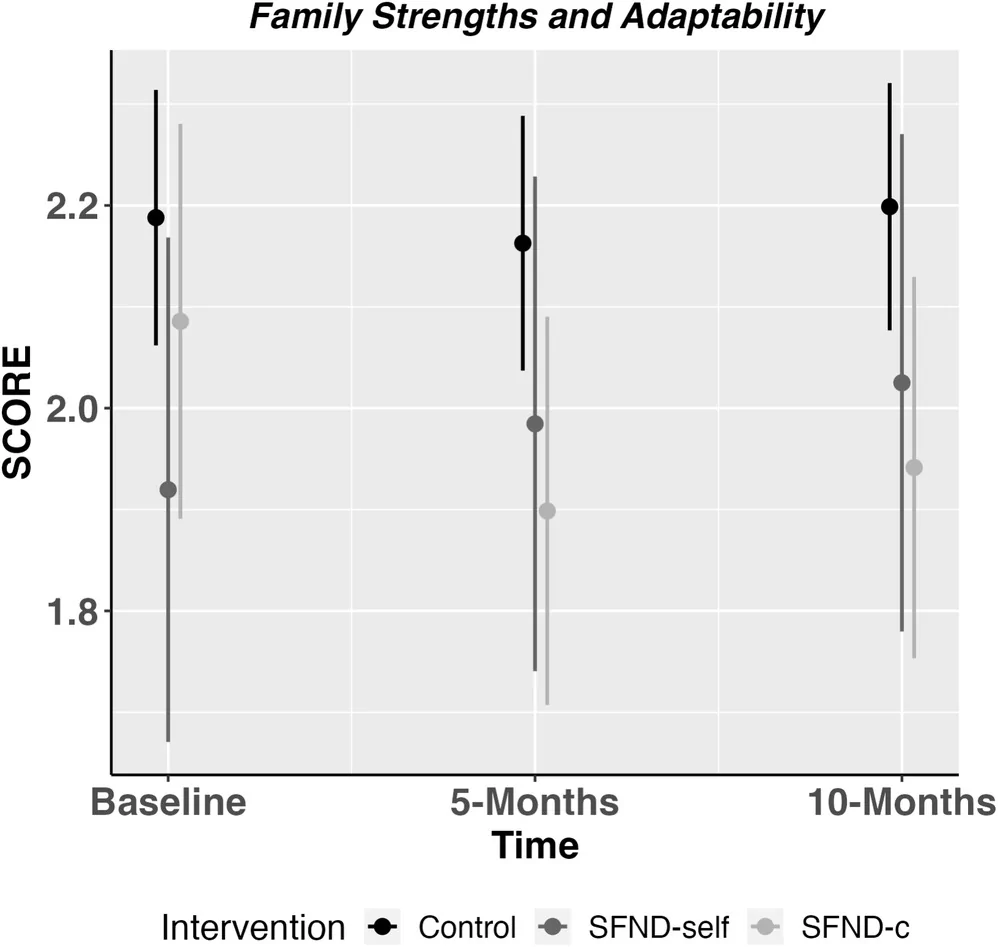
Plot of time*intervention interaction from interaction model for family strengths and adaptability subscale on family well‐being measure. Abbreviations: SFND‐c = strongest families neurodevelopmental—coached intervention; SFND‐self = strongest families neurodevelopmental—self‐directed intervention.

**FIGURE 4:**
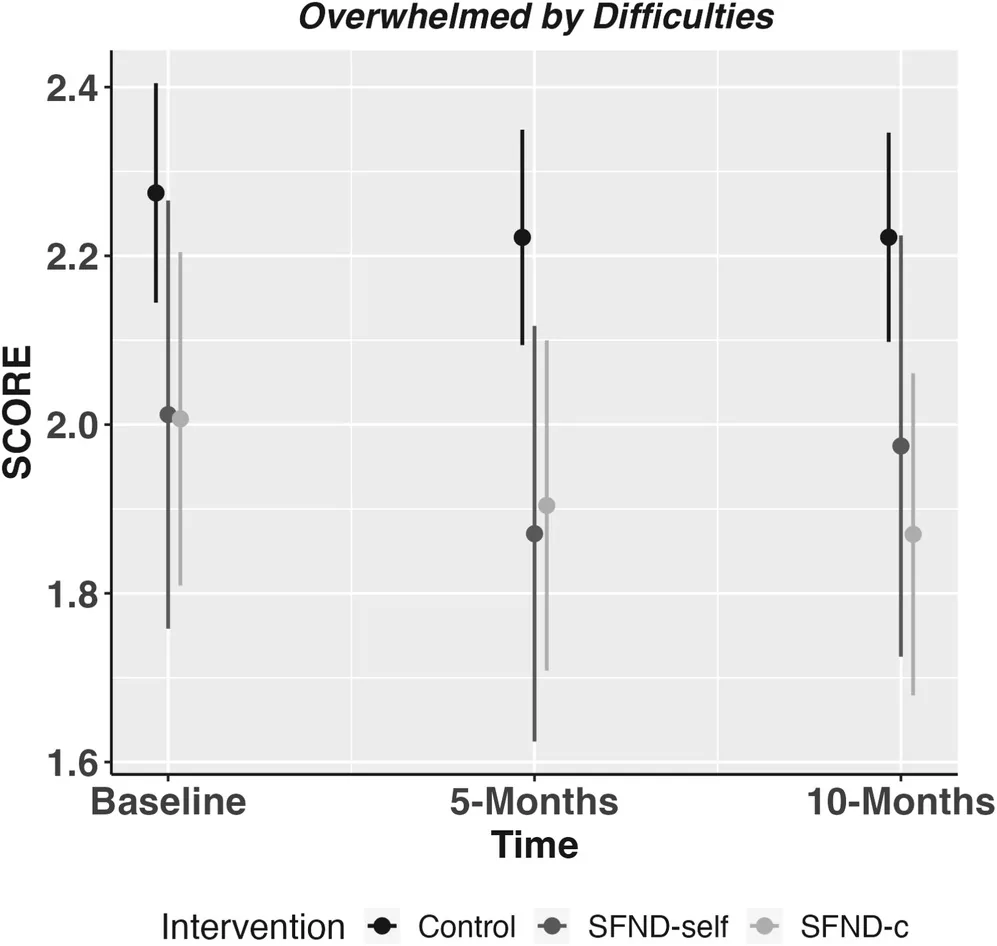
Plot of time*intervention interaction from interaction model for overwhelmed by difficulties subscale on family well‐being measure. Abbreviations: SFND‐c = strongest families neurodevelopmental—coached intervention; SFND‐self = strongest families neurodevelopmental—self‐directed intervention.

**FIGURE 5:**
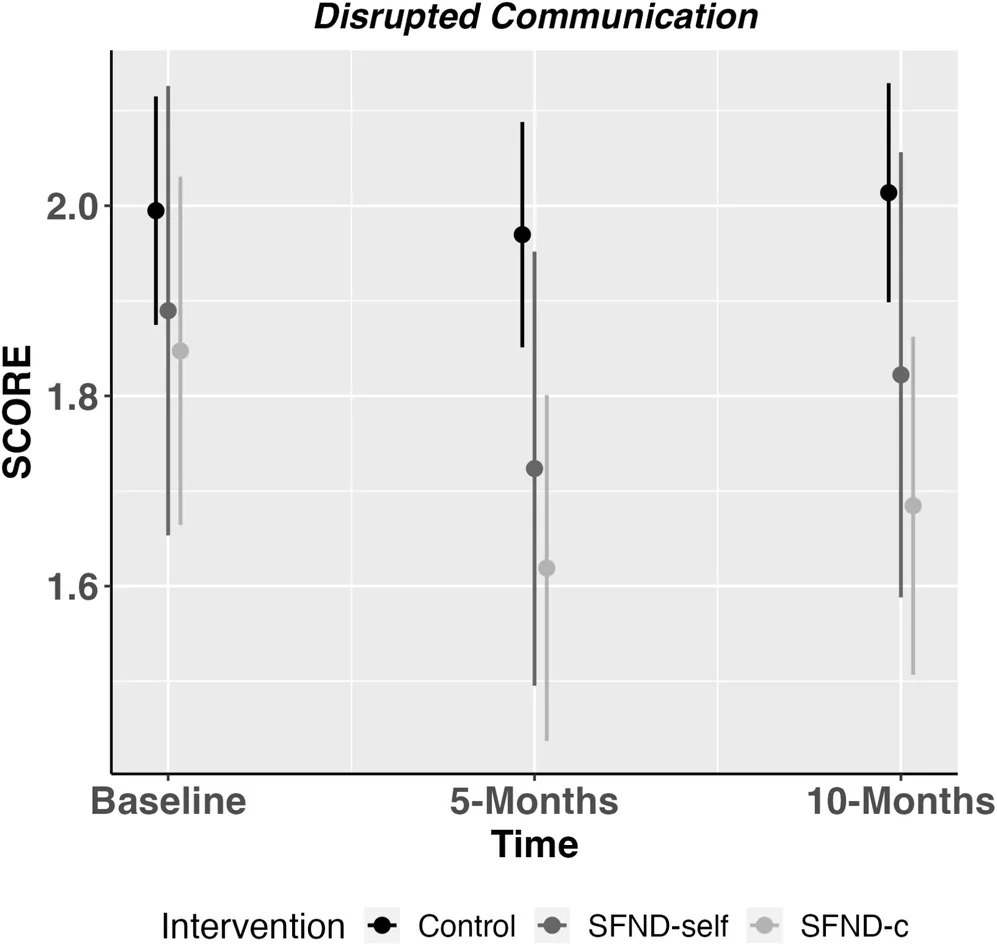
Plot of time*intervention interaction from interaction model for disrupted communication subscale on family well‐being measure. Abbreviations: SFND‐c = strongest families neurodevelopmental—coached intervention; SFND‐self = strongest families neurodevelopmental—self‐directed intervention.

To interpret the Interaction Model, an interaction term estimate that is significantly different from zero suggests that the effect of the intervention on the outcome is not constant over time. Our results indicated a significant interaction effect for the coached intervention at 5 months on the *Disrupted Communication* subscale (Table 4). No other interaction effects were noted, though the model suggests a trend toward significance for the coached model for several outcomes, including *Total Score* at 10 months, the *Family Strengths and Adaptability* subscale at both 5 and 10 months, as well as the *Disrupted Communication* subscale at 10 months (see Figures 2, 3, 4, 5 for time by intervention interaction plots).

**TABLE 4:** Coefficient estimate, confidence intervals, and *p* values for interaction model^b^.

Interaction model results			
Outcomes	Interventions	Estimates (95% CI)	*p* ^a^
Total scores	Baseline*SFND‐self	−3.05 (−6.64, 0.54)	0.098
	Baseline*SFND‐c	−2.79 (−5.79, 0.21)	0.069
	5 Months*SFND‐self	−0.88 (−3.68, 1.92)	0.538
	10 Months*SFND‐self	−0.24 (−3.12, 2.64)	0.873
	5 Months*SFND‐c	−1.85 (−4.20, 0.50)	0.124
	10 Months*SFND‐c	−1.99 (−4.30, 0.32)	0.094
Family strengths and adaptability	Baseline*SFND‐self	−0.27 (−0.54, 0.00)	0.060
	Baseline*SFND‐c	−0.10 (−0.34, 0.14)	0.387
	5 Months*SFND‐self	0.09 (−0.13, 0.31)	0.427
	10 Months*SFND‐self	0.09 (−0.13, 0.31)	0.411
	5 Months*SFND‐c	−0.16 (−0.34, 0.02)	0.086
	10 Months*SFND‐c	−0.16 (−0.34, 0.02)	0.098
Overwhelmed by difficulties	Baseline*SFND‐self	−0.26 (−0.55, 0.03)	0.071
	Baseline*SFND‐c	−0.27 (−0.51, −0.03)	0.027*
	5 Months*SFND‐self	−0.09 (−0.33, 0.15)	0.472
	10 Months*SFND‐self	0.02 (−0.23, 0.27)	0.903
	5 Months*SFND‐c	−0.05 (−0.25, 0.15)	0.628
	10 Months*SFND‐c	−0.08 (−0.28, 0.12)	0.408
Disrupted communications	Baseline*SFND‐self	−0.11 (−0.36, 0.14)	0.436
	Baseline*SFND‐c	−0.15 (−0.37, 0.07)	0.187
	5 Months*SFND‐self	−0.14 (−0.38, 0.10)	0.255
	10 Months*SFND‐self	−0.09 (−0.34, 0.16)	0.498
	5 Months*SFND‐c	−0.20 (−0.40, 0.00)	0.049*
	10 Months*SFND‐c	−0.18 (−0.38, 0.02)	0.075

Abbreviations: SFND‐c = strongest families neurodevelopmental—coached intervention; SFND‐self = strongest families neurodevelopmental—self‐directed intervention.

^a^ Significance levels: ***p < 0.001, **p < 0.01, *p < 0.05.

^b^ Outcome = change due to time + intervention + time•intervention interaction + residual.

We also developed a set of standard linear regression models that examined the average effect of the intervention across participants at the three timepoints without the random effects. The Basic Model demonstrated a significant difference between each of the SFND‐self and SFND‐c groups and the control group for the total score at both 5 and 10 months (Table 5) with 15%–23% of participants experiencing significant change on the total score for these time points as per the RCI calculations. This model also demonstrated highly significant (*p* < 0.01) differences between the SFND‐c and control group at both time points for nearly all subscales, as well as significance (*p* < 0.05) for the SFND‐self group for the *Overwhelmed by Difficulties* subscale at 5 months. These findings were mostly maintained with the addition of child‐centered covariates in the Child Model with additional significance noted for the SFND‐self group for the *Disrupted Communication* subscale at 5 months (Table S1). Significance was maintained for the SFND‐c group but not the SFND‐self group with the addition of parent‐centered covariates in the Child and Parent Model (Table S2). With the addition of family‐focused covariates in the Full Family Model, the SFND‐c group maintained a significant difference from the control group for the total score as well as the *Disrupted Communication* and *Overwhelmed by Difficulties* subscales at both time points (Table 6).

**TABLE 5:** Coefficient estimate, confidence intervals, and *p* values for the basic model for intervention at each time point^b^.

Basic model results				
Outcomes	Times	Interventions	Estimates (95% CI)	*p* ^a^
Total scores	Baseline	SFND‐self	−3.45 (−7.28, 0.39)	0.080
	Baseline	SFND‐c	−2.46 (−5.65, 0.74)	0.133
	5 Months	SFND‐self	−4.16 (−7.75, −0.57)	0.024*
	5 Months	SFND‐c	−6.00 (−9.05, −2.94)	0.000***
	10 Months	SFND‐self	−3.97 (−7.67, −0.28)	0.036*
	10 Months	SFND‐c	−4.88 (−7.84, −1.92)	0.001***
Family strengths and adaptability	Baseline	SFND‐self	−0.32 (−0.61, −0.04)	0.029*
	Baseline	SFND‐c	−0.10 (−0.34, 0.14)	0.397
	5 Months	SFND‐self	−0.21 (−0.49, 0.08)	0.154
	5 Months	SFND‐c	−0.35 (−0.58, −0.11)	0.004**
	10 Months	SFND‐self	−0.21 (−0.49, 0.08)	0.159
	10 Months	SFND‐c	−0.27 (−0.50, −0.04)	0.022*
Overwhelmed by difficulties	Baseline	SFND‐self	−0.26 (−0.55, 0.04)	0.089
	Baseline	SFND‐c	−0.21 (−0.46, 0.03)	0.090
	5 Months	SFND‐self	−0.36 (−0.63, −0.08)	0.011*
	5 Months	SFND‐c	−0.39 (−0.62, −0.16)	0.001***
	10 Months	SFND‐self	−0.27 (−0.56, 0.03)	0.077
	10 Months	SFND‐c	−0.35 (−0.59, −0.12)	0.004**
Disrupted communication	Baseline	SFND‐self	−0.11 (−0.40, 0.19)	0.481
	Baseline	SFND‐c	−0.12 (−0.37, 0.12)	0.321
	5 Months	SFND‐self	−0.25 (−0.49, 0.00)	0.053
	5 Months	SFND‐c	−0.42 (−0.63, −0.21)	0.000***
	10 Months	SFND‐self	−0.23 (−0.49, 0.02)	0.076
	10 Months	SFND‐c	−0.35 (−0.55, −0.14)	0.001***

Abbreviations: SFND‐c = strongest families neurodevelopmental—coached intervention; SFND‐self = strongest families neurodevelopmental—self‐directed intervention.

^a^ Significance levels: ***p < 0.001, **p < 0.01, *p < 0.05.

^b^ Outcome = change due to intervention + residual.

**TABLE 6:** Coefficient estimate, confidence intervals, and *p* values for the full family model for intervention at each time points^b^.

Full family model result				
Outcomes	Times	Interventions	Estimate (95% CI)	*p* ^a^
Total scores	Baseline	SFND‐self	−3.68 (−7.70, 0.35)	0.075
	Baseline	SFND‐c	−0.96 (−4.23, 2.30)	0.565
	5 Months	SFND‐self	−3.03 (−7.05, 0.99)	0.142
	5 Months	SFND‐c	−4.69 (−8.03, −1.36)	0.006**
	10 Months	SFND‐self	−3.72 (−7.77, 0.33)	0.073
	10 Months	SFND‐c	−4.30 (−7.44, −1.16)	0.008**
Family strengths and adaptability	Baseline	SFND‐self	−0.28 (−0.60, 0.03)	0.079
	Baseline	SFND‐c	−0.01 (−0.26, 0.24)	0.931
	5 Months	SFND‐self	−0.18 (−0.50, 0.14)	0.273
	5 Months	SFND‐c	−0.24 (−0.50, 0.02)	0.078
	10 Months	SFND‐self	−0.27 (−0.59, 0.05)	0.096
	10 Months	SFND‐c	−0.23 (−0.48, 0.02)	0.074
Overwhelmed by difficulties	Baseline	SFND‐self	−0.33 (−0.63, −0.02)	0.036*
	Baseline	SFND‐c	−0.11 (−0.36, 0.13)	0.358
	5 Months	SFND‐self	−0.27 (−0.57, 0.03)	0.080
	5 Months	SFND‐c	−0.30 (−0.55, −0.05)	0.020*
	10 Months	SFND‐self	−0.22 (−0.54, 0.09)	0.169
	10 Months	SFND‐c	−0.30 (−0.54, −0.05)	0.020*
Disrupted communication	Baseline	SFND‐self	−0.15 (−0.47, 0.17)	0.363
	Baseline	SFND‐c	−0.01 (−0.27, 0.24)	0.929
	5 Months	SFND‐self	−0.16 (−0.43, 0.12)	0.264
	5 Months	SFND‐c	−0.36 (−0.59, −0.13)	0.003**
	10 Months	SFND‐self	−0.13 (−0.41, 0.15)	0.348
	10 Months	SFND‐c	−0.31 (−0.53, −0.09)	0.006**

Abbreviations: SFND‐c = strongest families neurodevelopmental—coached intervention; SFND‐self = strongest families neurodevelopmental—self‐directed intervention.

^a^ Significance levels: ***p < 0.001, **p < 0.01, *p < 0.05.

^b^ Outcome = change due to intervention + child age + child gender + disability group + child function + behavior type + parent gender + parent age + parent education + additional family challenges + province of residence + residual.

No significant differences were found in the ANOVAs comparing the Basic Model to the Child Model. The Child and Parent Model demonstrated a significant improvement over the Basic Model only on the *Overwhelmed by Difficulties* subscale at baseline (*p* = 0.03). The Full Family Model yielded significant differences at baseline for the total score (*p* = 0.02) and the *Overwhelmed by Difficulties* subscale at baseline (*p* = 0.002) and 10 months (*p* = 0.03), suggesting an improvement in the model fit.

An RCI analysis was performed to evaluate whether the changes in individual scores were due to actual change rather than random variation or measurement error. Table 7 shows that 10%–28% of participants across various subscales and intervention groups experienced reliable significant changes during the study's follow‐up periods.

**TABLE 7:** Summary of reliable change index.

Summary of reliable change index calculations					
Intervention groups	Subscales	Follow‐up times	Total participants	# Significant change participants	Percentage significant changes
SFND‐c	Total score	5 Months	58	9	15.52
SFND‐c	Total score	10 Months	57	10	17.54
SFND‐c	Family strengths and adaptability	5 Months	58	7	12.07
SFND‐c	Family strengths and adaptability	10 Months	57	6	10.53
SFND‐c	Overwhelmed by difficulties	5 Months	58	16	27.59
SFND‐c	Overwhelmed by difficulties	10 Months	57	16	28.07
SFND‐c	Disrupted communication	5 Months	58	7	12.07
SFND‐c	Disrupted communication	10 Months	57	9	15.79
SFND‐self	Total score	5 Months	35	6	17.14
SFND‐self	Total score	10 Months	35	8	22.86
SFND‐self	Family strengths and adaptability	5 Months	35	8	22.86
SFND‐self	Family strengths and adaptability	10 Months	35	10	28.57
SFND‐self	Overwhelmed by difficulties	5 Months	35	5	14.29
SFND‐self	Overwhelmed by difficulties	10 Months	35	10	28.57
SFND‐self	Disrupted communication	5 Months	35	6	17.14
SFND‐self	Disrupted communication	10 Months	35	7	20.00

Abbreviations: SFND‐c = strongest families neurodevelopmental—coached intervention; SFND‐self = strongest families neurodevelopmental—self‐directed intervention.

## 4 Discussion

To our knowledge, this is the first large‐scale RCT examining family well‐being as a central outcome of an online parenting program designed to address behavioral challenges in children with conditions such as ASD, intellectual disability, cerebral palsy, and other brain‐based neurodisabilities. Previous reviews of the literature demonstrate that studies of parenting interventions for this population rarely examine family well‐being as an outcome, and those that have found limited or inconclusive effects (Dababnah and Parish 2016; Factor et al. 2019; Kuhaneck et al. 2015; McCrossin and Lach 2023b). The results of our exploratory analysis indicate significant improvements in aspects of family well‐being for those who participated in both versions of the online parenting program, with particularly robust findings for participants in the coached group. Our findings underscore the cascading benefits coached and self‐directed parenting interventions can have on the well‐being of the entire family system, moving beyond individual child and parent outcomes that are typically targeted by these programs.

The intervention, particularly the coached version, likely contributes to enhancing family well‐being by empowering parents to identify and understand patterns and contributing factors of family dynamics. This deeper comprehension allows parents to address issues more effectively, which subsequently improves communication within the family. Communication is indeed a commonly emphasized dimension of family functioning among other popular measures and corresponding theoretical models (Epstein et al. 2003; Olson 2000).

Several statistical approaches used in our data analysis demonstrate how the intervention influenced communication in families. In the Interaction Model the effect of intervention was significantly different (time by intervention interaction) at 5 months compared to baseline for the coached intervention for the *Disrupted Communication* subscale. Similar results were also trending toward significance at 10 months on the same subscale as well as for the *Family Strengths and Adaptability* subscale at both 5 and 10 months (Table 4). In the Basic Model, we found significant improvements for the coached but not the self‐directed version of the intervention on both the *Family Strengths and Adaptability* and *Disrupted Communication* subscales at both 5‐ and 10‐months post‐intervention (Table 5), findings which were partially maintained in the Full Family Model (Table 6). The significant findings across models suggest that the coaching element of the intervention may play a critical role in enhancing the effectiveness of the program. Given that many of the items within the *Family Strengths and Adaptability* subscale refer to positive communication skills (e.g., “In my family we talk to each other about things which matter to us” and “Each of us gets listened to in our family”) (Stratton et al. 2014), our results suggest that the components of the program that focus on improving communication within the family, such as shifting parental focus toward praising desirable behavior and improving parent skills in interpreting their child's behavior as communication, are likely enhanced through the coaching sessions.

Our comparative analysis revealed a superior model fit for the Full Family Model compared to the Basic Model. In addition to child and parent covariates, this model incorporated family‐level factors such as the presence of extenuating family circumstances affecting the family's capacity to care for their child (e.g., employment loss, caring for another member of the family with complex care needs). The improved fit for this model was particularly notable on the *Overwhelmed by Difficulties* subscale, underscoring the impact of considering the broader family circumstances—not just those of the individual child or parent—in order to fully understand the experiences of families.

This study's support for the efficacy of this parenting intervention aligns with family resilience theory and related previous research, which posits that targeted support for parents can foster resilience within families of children with neurodisabilities (Leone et al. 2016; McStay et al. 2014). The improvements observed in family well‐being, as measured by the SCORE‐15 (Stratton et al. 2014), suggest that such interventions can enhance the adaptive capacities of families, enabling them to navigate the complex challenges associated with neurodisabilities more effectively.

There are several strengths and limitations to our study. While we successfully recruited and randomized 454 families, a significant proportion did not complete a minimum threshold of the interventions to be included in the protocol analysis, leaving smaller samples for the self‐directed (*n* = 36) and coached (*n* = 60) intervention groups relative to the control group (*n* = 152). Baseline comparisons revealed that those who fully adhered began with significantly higher family well‐being scores—indicating not only that stronger initial functioning supports engagement, but also that a family well‐being screen at intake might help predict who will follow through with the program. Although this enhances the validity of our per‐protocol outcomes—by focusing on families who actually received the intervention—it also limits generalizability: families with lower baseline well‐being are less likely to benefit from the effects of the intervention.

An additional limitation is that the study took place amid the COVID‐19 pandemic, a time during which parents were managing children at home and reported having limited bandwidth to fully commit to the study. Future research should test whether targeted supports for families reporting lower well‐being and those with fewer resources can both improve adherence and extend benefits more equitably.

Furthermore, those who did not adhere to the protocol were less likely to report European ancestry—which may reflect cultural, linguistic, or contextual barriers to engagement. In addition, most of our participants identified as female caregivers of relatively high socioeconomic status, further limiting the generalizability of our findings. Future work should partner with under‐represented communities to co‐design and test culturally responsive adaptations, ensuring equitable access, improving adherence across diverse groups, and extending benefits to all families.

Lastly, the inclusion and exclusion criteria—and key baseline characteristics such as the child's diagnosis and the caregiver's own health status—relied on parent‐report, without independent clinical verification. This approach may have introduced reporting bias, and we cannot rule out that some participants were inaccurately categorized. Future studies should incorporate in‐person assessments or medical record reviews to confirm eligibility and enhance the validity of these important variables.

Despite these limitations, our results indicated a reliable change for 10%–28% of participants with particularly strong findings for the *Overwhelmed by Difficulties* subscale at 5 and 10 months, for the coached intervention (28%) and *Family Strengths and Adaptability* at 10 months, and for the self‐directed group (28%; Table 7). While exploratory, our results remain meaningful for a frontline, scalable parenting intervention.

## 5 Conclusions and Future Research

The impact of parenting interventions on family well‐being demonstrated in our study holds implications for clinical practice, policy, and research. Given the high incidence of behavioral difficulties co‐occurring with neurodisabilities and the systemic effects of these difficulties on the family unit, our results underscore the importance of offering accessible, cost‐effective, and scalable interventions to support these families. Programs like Parents Empowering Neurodiverse Kids not only address barriers faced by families in accessing conventional services, but also offer a promising, low‐cost approach to enhancing primary mental health support and bridging gaps between mental health and disability care.

Our finding that higher baseline family well‐being predicted greater adherence suggests that an initial family well‐being assessment could be used both to predict which families may struggle to engage and to tailor early supports—for example, booster sessions or peer mentoring—targeted at areas of greatest need. Embedding such an assessment at intake would help ensure that families who begin at a lower level of functioning receive the additional scaffolding required to fully benefit from the program.

Our findings prompt a deeper exploration of how child, parent, and family factors may mediate family outcomes, using sophisticated statistical methods such as structural equation modeling to achieve a more nuanced understanding of these relationships. Such approaches could continue to make use of family resilience theory, particularly for model specification (Gunty 2020; McCrossin and Lach 2023b). Future investigations should not only continue to dissect the “active ingredients” that specifically foster family well‐being, as opposed to individual child or parent outcomes, but also consider the cultural, systemic, and policy‐level frameworks that underpin the delivery of such programs. Adjustments to program content that emphasize principles of family‐centered care (Miller, Imms, et al. 2023; Trute and Hiebert‐Murphy 2013) may enhance the perceived relevance of the skills learned for the family unit, thereby improving family‐level outcomes.

## Funding

The first author was supported in part from funding from the Social Sciences and Humanities Research Council of Canada, and the Fonds de recherche du Québec. The study was funded by the Canadian Institutes of Health Research Support for Patient Oriented Research initiative—CHILD‐BRIGHT: Child Health Initiatives Limiting Disability—Brain Research Improving Growth and Health Trajectories (Grant #: SCA‐145104).

## Conflicts of Interest

The authors declare no conflicts of interest.

## Supporting information


**Table S1:** Coefficient estimate, confidence intervals, and *p* values for child model for intervention at each time point.^†^
**Table S2:** Coefficient estimate, confidence intervals, and *p* values for child and parent model for intervention at each time point.^†^
**Appendix I**. Model equations.

## Data Availability

The data that support the findings of this study are available on request from the corresponding author. The data are not publicly available due to privacy or ethical restrictions.

## References

[aur70334-bib-0001] Alderfer, M. A. , B. H. Fiese , J. I. Gold , et al. 2008. “Evidence‐Based Assessment in Pediatric Psychology: Family Measures.” Journal of Pediatric Psychology 33, no. 9: 1046–1061. 10.1093/jpepsy/jsm083. PMC2639492 17905801

[aur70334-bib-0002] Allard, A. , A. Fellowes , V. Shilling , A. Janssens , B. Beresford , and C. Morris . 2014. “Key Health Outcomes for Children and Young People With Neurodisability: Qualitative Research With Young People and Parents.” BMJ Open 4, no. 4: e004611. 10.1136/bmjopen-2013-004611. PMC3996811 24747792

[aur70334-bib-0003] Bailey, S. , L. Lach , and K. Byford‐Richardson . 2012. “Interpersonal Interactions and Relationships.” In Measures for Children With Developmental Disabilities: An Icf‐Cy Approach, edited by A. Majnemer , 381–403. Mac Keith Press.

[aur70334-bib-0004] Bearss, K. , C. Johnson , T. Smith , et al. 2015. “Effect of Parent Training vs Parent Education on Behavioral Problems in Children With Autism Spectrum Disorder: A Randomized Clinical Trial.” JAMA—Journal of the American Medical Association 313, no. 15: 1524–1533. 10.1001/jama.2015.3150. PMC9078140 25898050

[aur70334-bib-0005] Blampied, N. M. 2022. “Reliable Change and the Reliable Change Index: Still Useful After All These Years?” Cognitive Behaviour Therapist 15: e50. 10.1017/S1754470X22000484.

[aur70334-bib-0006] Carr, A. , and P. Stratton . 2017. “The SCORE Family Assessment Questionnaire: A Decade of Progress.” Family Process 56, no. 2: 285–301. 10.1111/famp.12280. 28205204

[aur70334-bib-0007] Chakraborty, H. , and H. Gu . 2009. A Mixed Model Approach for Intent‐To‐Treat Analysis in Longitudinal Clinical Trials With Missing Values. RTI International. 30896910

[aur70334-bib-0008] Dababnah, S. , and S. L. Parish . 2016. “A Comprehensive Literature Review of Randomized Controlled Trials for Parents of Young Children With Autism Spectrum Disorder.” Journal of Evidence‐Informed Social Work 13, no. 3: 277–292. 10.1080/23761407.2015.1052909. 26177069

[aur70334-bib-0009] Dekker, M. C. , H. M. Koot , J. van der Ende , and F. C. Verhulst . 2002. “Emotional and Behavioral Problems in Children and Adolescents With and Without Intellectual Disability.” Journal of Child Psychology and Psychiatry 43, no. 8: 1087–1098. 10.1111/1469-7610.00235. 12455929

[aur70334-bib-0010] Dykens, E. M. 2015. “Family Adjustment and Interventions in Neurodevelopmental Disorders.” Current Opinion in Psychiatry 28, no. 2: 121–126. 10.1097/YCO.0000000000000129. PMC5348480 25594421

[aur70334-bib-0011] Emerson, E. 2003. “Prevalence of Psychiatric Disorders in Children and Adolescents With and Without Intellectual Disability.” Journal of Intellectual Disability Research 47: 51–58. 10.1046/j.1365-2788.2003.00464.x. 12558695

[aur70334-bib-0012] Epstein, N. B. , L. M. Baldwin , and D. S. Bishop . 1983. “The McMaster Family Assessment Device.” Journal of Marital and Family Therapy 9, no. 2: 171–180. 10.1111/j.1752-0606.1983.tb01497.x.

[aur70334-bib-0013] Epstein, N. B. , C. E. Ryan , D. S. Bishop , I. W. Miller , and G. I. Keitner . 2003. “The McMaster Model: A View of Healthy Family Functioning.” In Normal Family Processes: Growing Diversity and Complexity, 3rd ed., edited by F. Walsh , 581–607. The Guilford Press. http://search.ebscohost.com.proxy‐ub.rug.nl/login.aspx?direct=true&db=psyh&AN=2003‐04409‐021&site=ehost‐live&scope=site.

[aur70334-bib-0014] Factor, R. S. , T. H. Ollendick , L. D. Cooper , J. C. Dunsmore , H. M. Rea , and A. Scarpa . 2019. “All in the Family: A Systematic Review of the Effect of Caregiver‐Administered Autism Spectrum Disorder Interventions on Family Functioning and Relationships.” Clinical Child and Family Psychology Review 22: 433–457. 10.1007/s10567-019-00297-x. 31363949

[aur70334-bib-0015] Garson, G. D. 2013. “Fundamentals of Hierarchical Linear and Multilevel Modeling.” In Hierarchical Linear Modeling: Guide and Applications, edited by G. D. Garson , 3–26. SAGE Publications, Inc.

[aur70334-bib-0016] Goodman, R. 2001. “Psychometric Properties of the Strengths and Difficulties Questionnaire.” Journal of the American Academy of Child and Adolescent Psychiatry 40: 1337–1345. 10.1097/00004583-200111000-00015. 11699809

[aur70334-bib-0017] Grenier‐Martin, J. , M. Rivard , S. Patel , M. J. Lanovaz , and C. Lefebvre . 2022. “Randomized Controlled Trial on an Online Training to Support Caregivers of Young Children With Intellectual and Developmental Disability Managing Problem Behaviors at Home.” Journal of Child and Family Studies 31: 3485–3497. 10.1007/s10826-022-02440-9.

[aur70334-bib-0018] Gunty, A. L. 2020. “Rethinking Resilience in Families of Children With Autism Spectrum Disorders.” Couple and Family Psychology: Research and Practice 10: 87–102. 10.1037/cfp0000155.

[aur70334-bib-0019] Hamilton, E. , A. Carr , P. Cahill , C. Cassells , and D. Hartnett . 2015. “Psychometric Properties and Responsiveness to Change of 15‐ and 28‐Item Versions of the SCORE: A Family Assessment Questionnaire.” Family Process 54, no. 3: 454–463. 10.1111/famp.12117. 25585671

[aur70334-bib-0020] Harris, P. A. , R. Taylor , R. Thielke , J. Payne , N. Gonzalez , and J. G. Conde . 2009. “Research Electronic Data Capture (REDCap)—A Metadata‐Driven Methodology and Workflow Process for Providing Translational Research Informatics Support.” Journal of Biomedical Informatics 42, no. 2: 377–381. 10.1016/j.jbi.2008.08.010. PMC2700030 18929686

[aur70334-bib-0021] Hoffman, L. , J. Marquis , D. Poston , J. A. Summers , and A. Turnbull . 2006. “Assessing Family Outcomes: Psychometric Evaluation of the Beach Center Family Quality of Life Scale.” Journal of Marriage and Family 68, no. 4: 1069–1083. 10.1111/j.1741-3737.2006.00314.x.

[aur70334-bib-0022] Jacobson, N. S. , and P. Truax . 1991. “Clinical Significance: A Statistical Approach to Denning Meaningful Change in Psychotherapy Research.” Journal of Consulting and Clinical Psychology 59, no. 1: 12–19. 10.1037//0022-006x.59.1.12 2002127

[aur70334-bib-0023] Kaminski, J. W. , L. A. Valle , J. H. Filene , and C. L. Boyle . 2008. “A Meta‐Analytic Review of Components Associated With Parent Training Program Effectiveness.” Journal of Abnormal Child Psychology 36, no. 4: 567–589. 10.1007/s10802-007-9201-9. 18205039

[aur70334-bib-0024] Karst, J. S. , and A. V. Van Hecke . 2012. “Parent and Family Impact of Autism Spectrum Disorders: A Review and Proposed Model for Intervention Evaluation.” Clinical Child and Family Psychology Review 15, no. 3: 247–277. 10.1007/s10567-012-0119-6. 22869324

[aur70334-bib-0025] Ketelaar, M. , A. Bogossian , M. Saini , A. Visser‐Meily , and L. Lach . 2017. “Assessment of the Family Environment in Pediatric Neurodisability: A State‐Of‐The‐Art Review.” Developmental Medicine and Child Neurology 59, no. 3: 259–269. 10.1111/dmcn.13287. 27696390

[aur70334-bib-0026] Kuhaneck, H. M. , S. Madonna , A. Novak , and E. Pearson . 2015. “Effectiveness of Interventions for Children With Autism Spectrum Disorder and Their Parents: A Systematic Review of Family Outcomes.” American Journal of Occupational Therapy 69, no. 5: 6905180040p1–6905180040p14. 10.5014/ajot.2015.017855. 26356656

[aur70334-bib-0027] Lach, L. M. 2013. “The Family Does Matter!” In Life Quality Outcomes in Children and Young People With Neurological and Developmental Conditions: Concepts, Evidence and Practice, edited by G. M. Ronen and P. L. Rosenbaum , 136–153. MacKeith Press.

[aur70334-bib-0028] Lach, L. M. , D. E. Kohen , R. E. Garner , et al. 2009. “The Health and Psychosocial Functioning of Caregivers of Children With Neurodevelopmental Disorders.” Disability and Rehabilitation 31, no. 9: 741–752. 10.1080/08916930802354948. 19736648

[aur70334-bib-0029] Leone, E. , D. Dorstyn , and L. Ward . 2016. “Defining Resilience in Families Living With Neurodevelopmental Disorder: A Preliminary Examination of Walsh's Framework.” Journal of Developmental and Physical Disabilities 28, no. 4: 595–608. 10.1007/s10882-016-9497-x.

[aur70334-bib-0030] Lingely‐Pottie, P. , and P. McGrath . 2023. “Paraprofessional Roles in e‐Mental Health.” Psynopsis 45, no. 2: 31–32.

[aur70334-bib-0031] Lingley‐Pottie, P. , and P. McGrath . 2016. “Imagine a Mental Health Service That Builds Stronger Families.” Paediatrics and Child Health 21, no. 5: 247–248. 10.1093/pch/21.5.247. PMC4933053 27441017

[aur70334-bib-0032] Masten, A. S. , and A. R. Palmer . 2019. “Parenting to Promote Resilience in Children.” In Handbook of Parenting, edited by M. H. Bornstein , 3rd ed., 156–188. Routledge. 10.4324/9780429401695-6.

[aur70334-bib-0033] Maurović, I. , L. Liebenberg , and M. Ferić . 2020. “A Review of Family Resilience: Understanding the Concept and Operationalization Challenges to Inform Research and Practice.” Child Care in Practice 26, no. 4: 337–357. 10.1080/13575279.2020.1792838.

[aur70334-bib-0034] McCrossin, J. , and L. Lach . 2023a. “Parent‐to‐Parent Support for Childhood Neurodisability: A Qualitative Analysis and Proposed Model of Peer Support and Family Resilience.” Child: Care, Health and Development 49, no. 3: 544–554. 10.1111/cch.13069. 36222028

[aur70334-bib-0035] McCrossin, J. , L. Lach , and P. McGrath . 2023. “Content Analysis of Parent Training Programs for Children With Neurodisabilities and Mental Health or Behavioral Problems: A Scoping Review.” Disability and Rehabilitation 45, no. 1: 154–169. 10.1080/09638288.2021.2017493. 34990567

[aur70334-bib-0036] McCrossin, J. , and L. M. Lach . 2023b. “Measuring Family Outcomes in Parenting Programs for Children With Neurodisabilities: A Scoping Review.” Journal of Family Social Work 26, no. 2: 68–101. 10.1080/10522158.2023.2231986.

[aur70334-bib-0037] McCubbin, H. I. , and J. M. Patterson . 1983. “The Family Stress Process: The Double Abcx Model of Adjustment and Adaptation.” Marriage and Family Review 6, no. 1–2: 7–37. 10.1300/J002v06n01_02.

[aur70334-bib-0038] McGrath, P. J. , P. Lingley‐Pottie , C. Thurston , et al. 2011. “Telephone‐Based Mental Health Interventions for Child Disruptive Behavior or Anxiety Disorders: Randomized Trials and Overall Analysis.” Journal of the American Academy of Child and Adolescent Psychiatry 50, no. 11: 1162–1172. 10.1016/j.jaac.2011.07.013. 22024004

[aur70334-bib-0039] McStay, R. L. , D. Trembath , and C. Dissanayake . 2014. “Maternal Stress and Family Quality of Life in Response to Raising a Child With Autism: From Preschool to Adolescence.” Research in Developmental Disabilities 35, no. 11: 3119–3130. 10.1016/j.ridd.2014.07.043 25145805

[aur70334-bib-0040] Micai, M. , L. M. Fatta , L. Gila , et al. 2023. “Prevalence of Co‐Occurring Conditions in Children and Adults With Autism Spectrum Disorder: A Systematic Review and Meta‐Analysis.” Neuroscience and Biobehavioral Reviews 155: 105436. 10.1016/j.neubiorev.2023.105436. 37913872

[aur70334-bib-0041] Miller, A. , E. Gardiner , and P. L. Rosenbaum . 2023. “A Non‐Categorical Approach to Childhood Neurodisability: Concepts, Evidence, and Implications for Clinical Practice, Organization of Services, Teaching, and Research.” In Neurodevelopmental Pediatrics: Genetic and Environmental Influences, edited by D. D. Eisenstat , D. Goldowitz , T. F. Oberlander , and J. Y. Yager , 685–695. Springer International Publishing. 10.1007/978-3-031-20792-1_42.

[aur70334-bib-0042] Miller, L. , C. Imms , A. Cross , et al. 2023. “Impact of “Early Intervention” Parent Workshops on Outcomes for Caregivers of Children With Neurodisabilities: A Mixed‐Methods Study.” Disability and Rehabilitation 45, no. 23: 3900–3911. 10.1080/09638288.2022.2143579. 36404703

[aur70334-bib-0043] Morris, C. , A. Janssens , R. Tomlinson , J. Williams , and S. Logan . 2013. “Towards a Definition of Neurodisability: A Delphi Survey.” Developmental Medicine and Child Neurology 55, no. 12: 1103–1108. 10.1111/dmcn.12218. 23909744

[aur70334-bib-0044] Olson, D. H. 2000. “Circumplex Model of Marital and Family Systems.” Journal of Family Therapy 22, no. 2: 144–167.

[aur70334-bib-0045] Olthuis, J. V. , P. J. McGrath , C. E. Cunningham , et al. 2018. “Distance‐Delivered Parent Training for Childhood Disruptive Behavior (Strongest Families): A Randomized Controlled Trial and Economic Analysis.” Journal of Abnormal Child Psychology 46, no. 8: 1613–1629. 10.1007/s10802-018-0413-y. 29516341

[aur70334-bib-0046] Peters, S. A. E. , M. L. Bots , H. M. den Ruijter , et al. 2012. “Multiple Imputation of Missing Repeated Outcome Measurements Did Not Add to Linear Mixed‐Effects Models.” Journal of Clinical Epidemiology 65, no. 6: 686–695. 10.1016/j.jclinepi.2011.11.012. 22459429

[aur70334-bib-0047] Petrenko, C. L. M. 2013. “A Review of Intervention Programs to Prevent and Treat Behavioral Problems in Young Children With Developmental Disabilities.” Journal of Developmental and Physical Disabilities 25, no. 6: 651–679. 10.1007/s10882-013-9336-2. PMC3821779 24222982

[aur70334-bib-0048] Petrou, A. M. , A. Soul , B. Koshy , H. McConachie , and J. R. Parr . 2018. “The Impact on the Family of the Co‐Existing Conditions of Children With Autism Spectrum Disorder.” Autism Research 11, no. 5: 776–787. 10.1002/aur.1932. 29427538

[aur70334-bib-0049] Postorino, V. , W. G. Sharp , C. E. McCracken , et al. 2017. “A Systematic Review and Meta‐Analysis of Parent Training for Disruptive Behavior in Children With Autism Spectrum Disorder.” Clinical Child and Family Psychology Review 20, no. 4: 391–402. 10.1007/s10567-017-0237-2. 28600643

[aur70334-bib-0050] Sanders, M. R. , G. Divan , M. Singhal , et al. 2021. “Scaling Up Parenting Interventions Is Critical for Attaining the Sustainable Development Goals.” Child Psychiatry and Human Development 53: 941–952. 10.1007/s10578-021-01171-0. PMC8096135 33948778

[aur70334-bib-0051] Sikora, D. , E. Moran , F. Orlich , et al. 2013. “The Relationship Between Family Functioning and Behavior Problems in Children With Autism Spectrum Disorders.” Research in Autism Spectrum Disorders 7, no. 2: 307–315. 10.1016/j.rasd.2012.09.006.

[aur70334-bib-0052] Sourander, A. , P. J. McGrath , T. Ristkari , et al. 2018. “Two‐Year Follow‐Up of Internet and Telephone Assisted Parent Training for Disruptive Behavior at Age 4.” Journal of the American Academy of Child and Adolescent Psychiatry 57, no. 9: 658–668.e1. 10.1016/j.jaac.2018.07.001. 30196869

[aur70334-bib-0053] Sourander, A. , P. J. McGrath , T. Ristkari , et al. 2016. “Internet‐Assisted Parent Training Intervention for Disruptive Behavior in 4‐Year‐Old Children: A Randomized Clinical Trial.” JAMA Psychiatry 73, no. 4: 378. 10.1001/jamapsychiatry.2015.3411. 26913614

[aur70334-bib-0054] Stratton, P. , J. Lask , J. Bland , et al. 2014. “Detecting Therapeutic Improvement Early in Therapy: Validation of the SCORE‐15 Index of Family Functioning and Change.” Journal of Family Therapy 36, no. 1: 3–19. 10.1111/1467-6427.12022.

[aur70334-bib-0055] Tarver, J. , M. Palmer , S. Webb , et al. 2019. “Child and Parent Outcomes Following Parent Interventions for Child Emotional and Behavioral Problems in Autism Spectrum Disorders: A Systematic Review and Meta‐Analysis.” Autism 23, no. 7: 1630–1644. 10.1177/1362361319830042. 30818965

[aur70334-bib-0056] Trute, B. , and D. Hiebert‐Murphy . 2013. Partnering With Parents: Family‐Centred Practice in Children's Services. University of Toronto Press. https://utorontopress.com/ca/partnering‐with‐parents‐4.

[aur70334-bib-0057] Ungar, M. 2010. “Families as Navigators and Negotiators: Facilitating Culturally and Contextually Specific Expressions of Resilience.” Family Process 49, no. 3: 421–435. 10.1111/j.1545-5300.2010.01331.x. 20831769

[aur70334-bib-0058] Ungar, M. 2019. “Designing Resilience Research: Using Multiple Methods to Investigate Risk Exposure, Promotive and Protective Processes, and Contextually Relevant Outcomes for Children and Youth.” Child Abuse and Neglect 96: 104098. 10.1016/j.chiabu.2019.104098. 31376582

[aur70334-bib-0059] Walsh, F. 2021. “Family Resilience: A Dynamic Systemic Framework.” In Multisystemic Resilience. Adaptation and Transformation in Contexts of Change, edited by M. Ungar , 255–270. Oxford University Press. 10.1093/oso/9780190095888.003.0015.

[aur70334-bib-0060] Weeland, J. , K. O. W. Helmerhorst , and N. Lucassen . 2021. “Understanding Differential Effectiveness of Behavioral Parent Training From a Family Systems Perspective: Families Are Greater Than ‘Some of Their Parts.’.” Journal of Family Theory and Review 13, no. 1: 34–57. 10.1111/jftr.12408.

[aur70334-bib-0061] Women and Children's Health Research Institute . 2022. “Who We Are.” https://www.wchri.org/about‐wchri/who‐we‐are/.

