# Supplementary material for: The Effect of Online Parent Training With and Without Coaching Support on the Well‐Being of Families of Children With Neurodisabilities: A Randomized Controlled Trial
Source: Autism Res. 2026 Jul 30;19(9):e70334. doi: 10.1002/aur.70334 (PMC13599695; doi:10.1002/aur.70334)
Supplement: Supplementary file 1 [file AUR-19-0-s001.docx]

# Supplementary Tables

### Supplementary Table 1

##### Coefficient estimate, confidence intervals, and P-values for Child Model for intervention at each time point.^†^

| Child Model Results | | | | |
| --- | --- | --- | --- | --- |
| Outcome | Time | Intervention | Estimate (95% CI) | P-Value*^1^* |
| Total Score | Baseline | SFND-self | -3.54 (-7.39, 0.32) | 0.074 |
|  | Baseline | SFND-c | -1.40 (-4.72, 1.92) | 0.409 |
|  | 5 Months | SFND-self | -4.41 (-8.08, -0.74) | 0.020* |
|  | 5 Months | SFND-c | -5.66 (-8.87, -2.45) | 0.001*** |
|  | 10 Months | SFND-self | -4.14 (-7.91, -0.36) | 0.033* |
|  | 10 Months | SFND-c | -4.42 (-7.50, -1.35) | 0.005** |
| Family Strengths and Adaptability | Baseline | SFND-self | -0.32 (-0.62, -0.03) | 0.031* |
|  | Baseline | SFND-c | -0.04 (-0.29, 0.21) | 0.777 |
|  | 5 Months | SFND-self | -0.21 (-0.50, 0.07) | 0.143 |
|  | 5 Months | SFND-c | -0.30 (-0.55, -0.06) | 0.017* |
|  | 10 Months | SFND-self | -0.21 (-0.51, 0.08) | 0.154 |
|  | 10 Months | SFND-c | -0.22 (-0.46, 0.02) | 0.072 |
| Overwhelmed by Difficulties | Baseline | SFND-self | -0.28 (-0.58, 0.01) | 0.064 |
|  | Baseline | SFND-c | -0.15 (-0.40, 0.10) | 0.249 |
|  | 5 Months | SFND-self | -0.38 (-0.66, -0.11) | 0.007** |
|  | 5 Months | SFND-c | -0.36 (-0.60, -0.11) | 0.004** |
|  | 10 Months | SFND-self | -0.30 (-0.60, 0.00) | 0.054 |
|  | 10 Months | SFND-c | -0.33 (-0.58, -0.08) | 0.009** |
| Disrupted Communication | Baseline | SFND-self | -0.11 (-0.41, 0.19) | 0.481 |
|  | Baseline | SFND-c | -0.05 (-0.30, 0.20) | 0.703 |
|  | 5 Months | SFND-self | -0.26 (-0.51, -0.00) | 0.048* |
|  | 5 Months | SFND-c | -0.42 (-0.64, -0.20) | 0.000*** |
|  | 10 Months | SFND-self | -0.23 (-0.49, 0.03) | 0.087 |
|  | 10 Months | SFND-c | -0.32 (-0.54, -0.11) | 0.003** |
| ^1^ Significance levels: *** p<0.001, ** p<0.01, * p<0.05. *SFND-c = Strongest Families Neurodevelopmental – coached intervention; SFND-self = Strongest Families Neurodevelopmental – self-directed intervention*  † Outcome = change due to intervention + child age + child gender + disability group + child function + behaviour type + residual | | | | |

### Supplementary Table 2

##### Coefficient estimate, confidence intervals, and P-values for Child and Parent Model for intervention at each time point.^†^

| Parent and Child Model Results | | | | |
| --- | --- | --- | --- | --- |
| Outcome | Time | Intervention | Estimate (95% CI) | P-Value1 |
| Total Score | Baseline | SFND-self | -2.72 (-6.68, 1.24) | 0.181 |
|  | Baseline | SFND-c | -1.28 (-4.59, 2.04) | 0.451 |
|  | 5 Months | SFND-self | -3.16 (-7.03, 0.71) | 0.111 |
|  | 5 Months | SFND-c | -4.85 (-8.14, -1.56) | 0.004** |
|  | 10 Months | SFND-self | -3.71 (-7.70, 0.28) | 0.070 |
|  | 10 Months | SFND-c | -4.17 (-7.30, -1.03) | 0.010* |
| Family Strengths and Adaptability | Baseline | SFND-self | -0.26 (-0.56, 0.04) | 0.094 |
|  | Baseline | SFND-c | -0.02 (-0.27, 0.23) | 0.857 |
|  | 5 Months | SFND-self | -0.14 (-0.44, 0.17) | 0.383 |
|  | 5 Months | SFND-c | -0.26 (-0.51, -0.00) | 0.050 |
|  | 10 Months | SFND-self | -0.24 (-0.55, 0.07) | 0.134 |
|  | 10 Months | SFND-c | -0.22 (-0.47, 0.03) | 0.081 |
| Overwhelmed by Difficulties | Baseline | SFND-self | -0.25 (-0.55, 0.05) | 0.108 |
|  | Baseline | SFND-c | -0.13 (-0.38, 0.12) | 0.305 |
|  | 5 Months | SFND-self | -0.32 (-0.61, -0.02) | 0.035* |
|  | 5 Months | SFND-c | -0.30 (-0.55, -0.05) | 0.019* |
|  | 10 Months | SFND-self | -0.23 (-0.55, 0.08) | 0.140 |
|  | 10 Months | SFND-c | -0.30 (-0.54, -0.05) | 0.019* |
| Disrupted Communication | Baseline | SFND-self | -0.05 (-0.37, 0.26) | 0.741 |
|  | Baseline | SFND-c | -0.04 (-0.30, 0.22) | 0.750 |
|  | 5 Months | SFND-self | -0.17 (-0.43, 0.10) | 0.217 |
|  | 5 Months | SFND-c | -0.37 (-0.60, -0.15) | 0.001*** |
|  | 10 Months | SFND-self | -0.17 (-0.44, 0.11) | 0.237 |
|  | 10 Months | SFND-c | -0.30 (-0.52, -0.08) | 0.007** |
| 1 Significance levels: *** p<0.001, ** p<0.01, * p<0.05. *SFND-c = Strongest Families Neurodevelopmental – coached intervention; SFND-self = Strongest Families Neurodevelopmental – self-directed intervention*  † Outcome = change due to intervention + child age + child gender + disability group + child function + behaviour type + parent gender + parent age + parent education + residual | | | | |

# Appendix I

## Model Equations

### Interaction Model

###

$$\boldsymbol{y}_{\boldsymbol{ij}}\mathbf{=}\boldsymbol{\beta}_{\mathbf{0}}\mathbf{+}\boldsymbol{\beta}_{\mathbf{1}}\boldsymbol{\cdot}\text{time}_{\boldsymbol{ij}}\mathbf{+}\boldsymbol{\beta}_{\mathbf{2}}\boldsymbol{\cdot}\text{intervention}_{\boldsymbol{ij}}\mathbf{+}\boldsymbol{\beta}_{\mathbf{3}}\boldsymbol{\cdot}\left( \text{time}_{\boldsymbol{ij}}\boldsymbol{\cdot}\text{intervention}_{\boldsymbol{ij}} \right)\mathbf{+}\boldsymbol{u}_{\boldsymbol{j}}\mathbf{+}\boldsymbol{\epsilon}_{\boldsymbol{ij}}$$

### (1)

Where

$y_{ij}$ is the outcome for the i-th observation of the j-th subject.

$\beta_{0}$ is the overall intercept (the average outcome when time and intervention are at their reference levels).

$\beta_{1}$ is the fixed effect coefficient for time, indicating how much outcome is expected to change with each unit of time, assuming all other variables are held constant.

$\beta_{2}$ is the fixed effect coefficient for intervention.

$\beta_{3}$ is the time by intervention interaction coefficient.

$\boldsymbol{\epsilon}_{\boldsymbol{ij}}$ is the residual

### Basic Model

###

$$\boldsymbol{y=}\boldsymbol{\beta}_{\boldsymbol{0}}\boldsymbol{+}\boldsymbol{\beta}_{\boldsymbol{1}}\boldsymbol{\cdot}\text{intervention}\boldsymbol{+\in}$$

### (2.1)

### Child Model

$$y=\beta_{0}+\beta_{1}\cdot\text{intervention}+\beta_{2}\cdot\text{child age}+\beta_{3}\times\text{child gender}$$

$$+\beta_{4}\cdot\text{disability group}+\beta_{5}\cdot\text{child function}+\beta_{6}\cdot\text{behaviour type }+ \in$$

### (2.2)

### Parent Model

$$y=\beta_{0}+\beta_{1}\cdot\text{intervention}+\beta_{2}\cdot\text{child age}+\beta_{3}\cdot\text{child gender}$$

$$+\beta_{4}\cdot\text{disability group}+\beta_{5}\cdot\text{child function}+\beta_{6}\cdot\text{behaviour type}$$

$$+\beta_{7}\cdot\text{parent gender}+\beta_{8}\cdot\text{parent age}+\beta_{9}\cdot\text{parent education }+ \in$$

### (2.3)

### Full Family Model

$$y=\beta_{0}+\beta_{1}\cdot\text{intervention}+\beta_{2}\cdot\text{child age}+\beta_{3}\cdot\text{child gender}$$

$$+\beta_{4}\cdot\text{disability group}+\beta_{5}\cdot\text{child function}+\beta_{6}\cdot\text{behaviour type}$$

$$+\beta_{7}\cdot\text{parent gender}+\beta_{8}\cdot\text{parent age}+\beta_{9}\cdot\text{parent education}$$

$$+\beta_{10}\cdot\text{additional family challenges}+\beta_{11}\cdot\text{province of residence }+ \in$$

### (2.4)

Where

$y$ is the outcome.

$\beta_{0}$ is the overall intercept.

$\beta_{1}$ is the coefficient for intervention.

$\beta_{2-11}$ are the coefficients for child, parent, and family-level covariates.
